# Insights into the origins of inverted circular dichroism in thin films of a chiral side chain polyfluorene

**DOI:** 10.1002/chir.23601

**Published:** 2023-06-22

**Authors:** Louis Minion, Jessica Wade, Juan Manuel Moreno‐Naranjo, Seán Ryan, Giuliano Siligardi, Matthew J. Fuchter

**Affiliations:** ^1^ Department of Materials Imperial College London London UK; ^2^ Centre for Processable Electronics Imperial College London, South Kensington Campus London UK; ^3^ B23 Beamline, Diamond Light Source Ltd, Harwell Science and Innovation Campus Didcot UK; ^4^ Department of Chemistry and Molecular Sciences Research Hub Imperial College London, White City Campus London UK

**Keywords:** circular dichroism, conjugated polymer, copolymers, optical activity, polyfluorene, polythiophene

## Abstract

We synthesized a fluorene‐bithiophene co‐polymer with chiral side chains (**cPFT2**) and investigated its chiroptical properties via synchotronradiation circular dichroism. We observed that thin films of the polymer display an intense circular dichroism (CD) upon annealing, which is of opposite handedness to the CD reported for similar polyfluorenes bearing the same enantiomeric chiral side chain. We then contrast the properties of this polymer with chiral side chain fluorene homopolymer (**cPF**) and observe large differences in their thin film morphology. Using photoluminescence spectroscopy, we uncover evidence of polymer chain bending in **cPFT2**, which is further supported by theoretical calculations, and propose an explanation for the observed inverted optical activity.

## Graphical abstract

Chiral side chain fluorene (cPF) and fluorene‐bithiophene (cPFT2) co‐polymers display intense CD upon annealing. Despite having the same handedness of enantiopure side chain (here (*S*)‐), the sign of the lowest energy circular dichroism is opposite for the two polymers. We combine microscopy, spectroscopy, and computational simulations to propose that chain bending in the more flexible fluorene‐bithiophene co‐polymer is responsible for this inversion.

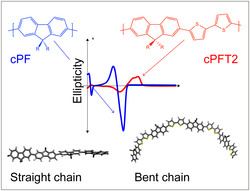

**Abbreviations**
AFM: Atomic Force Microscopy
CD: circular dichroism
F8T2: poly(9,9‐dioctylfluorene‐alt‐bithiophene)
PFO: poly(9,9‐dioctylfluorene)
PL: photoluminescence

## 1 INTRODUCTION

To accelerate and realize the application of conjugated organic polymers in real‐world technologies it is critical to understand and optimize their optical and electronic properties. Recently, molecular chirality has emerged as a strategy to impart new functionality into the active layers of optoelectronic devices. Optically active chiral materials will find application in high‐efficiency displays and circularly selective photodetectors, while the chiral organization of conjugated organic molecules can give rise to spin‐selective charge transport due to the chirality‐induced spin selectivity effect.^1^, ^2^, ^3^

Several strategies have emerged to introduce chirality into polymer thin films, including the incorporation of chiral side chains, the combination of achiral polymers with chiral small molecule additives, and the use of chiral solvents.^4^, ^5^, ^6^, ^7^, ^8^ The molecular weight, processing conditions, and post‐deposition treatments (e.g., thermal annealing, solvent vapor exposure) have been shown to play a critical role in determining the strength of the optical response.^9^, ^10^, ^11^, ^12^ While design rules are still emerging, polyfluorene derivatives have far outperformed other conjugated polymers.^13^, ^14^, ^15^, ^16^, ^17^

In particular, poly(9,9‐dioctyl‐fluorene‐*alt*‐bithiophene) (F8T2)—a polymer developed for organic light emitting diodes (OLEDs) and phototransistors^18^, ^19^—has shown large chiroptical responses after aggregation in chiral solvents (e.g., limonene^20^) and on blending with chiral small molecules (e.g., aza[6]helicene^21^, ^22^, ^23^, ^24^). The precise molecular packing that gives rise to the giant chiroptical response in chiral phases of F8T2 is not well understood, which is surprising given the range of helical conformations that have been previously attributed to chiral side chain polythiophenes.^25^, ^26^

**FIGURE 1:**
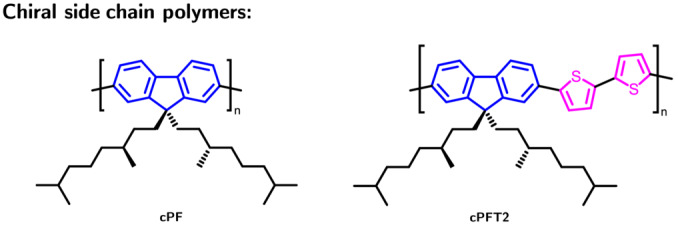
Two chiral polyfluorene analogs were investigated, cPF and cPFT2, with chemical structures shown here.

Here, we use side chain engineering to introduce chirality into an F8T2 analog (cPFT2, Figure 1). While similar chemical structures have been considered in the literature, thin film fabrication has not been optimized and their optical response not carefully scrutinized.^5^, ^21^, ^27^, ^28^ For example, annealed thin films of chiral side chain polymers containing fluorene and thiophene units display a positive bisignate cotton effect,^21^, ^27^ which is surprising given the handedness of the enantiopure side chains employed in these studies (e.g., (*S*)). With a fixed chiral side chain ((*S*)‐3,7‐dimethyloctyl), we contrast the optical activity of cPFT2 with the well‐studied homopolymer chiral side chain polyfluorene (cPF). The two polymers display opposite sign bisignate CD spectra. Atomic force microscopy reveals that annealed thin films of the two polymers adopt a strikingly different morphology: the surface of cPF comprises a dense network of short, twisted fibril‐like structures, while cPFT2 contains an assembly of smooth, curved, connected domains. Our results indicate that chiral aggregation of conjugated polymers is not only determined by the handedness of the chiral side chain (or chiral small molecule additive additive) but also depends on the conjugation length and torsional flexibility of the main chain. Uncovering the origin of these differences is vital for understanding and exploiting structure–property relationships in chiral polymer systems.

## 2 MATERIALS AND METHODS

### 2.1 Chemicals and synthesis

Details of the synthesis of cPF have been provided elsewhere.^22^ To prepare cPFT2, 2,7‐bis(4,4,5,5‐tetramethyl‐1,3,2‐dioxaborolan‐2‐yl)‐9,9‐bis[(3S)‐3, 7‐dimethyloctyl]‐9H‐fluorene (cF8Bpin, 482 mg, 0.69 mmol, 1.00 equiv), 5,5*′*‐dibromo‐2,2*′*‐bithiophene (224 mg, 0.69 mmol, 1.0 equiv), Pd(PPh_3_)_4_ (tetrakis(triphenylphosphine)‐palladium(0), 12 mg, 0.01 mmol, 0.015 equiv) and aliquote (trioctylmethylammonium chloride, 15 mg) were added to a 25mL two‐neck round bottomed flask with a stirrer bar and reflux condenser and placed under a nitrogen atmosphere. Nitrogen‐purged 2M NaOH (sodium hydroxide, 3 mL) and toluene (15 mL) were added to the reaction vessel and the mixture was subjected to three freeze‐pump‐thaw cycles. After 60 h, a solution of cF8Bpin (48 mg, 0.07 mmol, 0.10 equiv) and Pd(PPh_3_)_4_ (1.2 mg, 0.001 mmol, 0.001 equiv) in toluene (2 mL) was deoxygenated by sparging with nitrogen for 10 min and added to the reaction mixture. After an additional 4 h at 100°C, a solution of PhI (iodobenzene, 0.5 mL) and Pd(PPh_3_)_4_ (1.2 mg) in toluene (2 mL) was deoxygenated by sparging with nitrogen for 10 min and added to the reaction mixture. After an additional 4 h at 100°C, the reaction was allowed to cool to room temperature and diethyldithiocarbamate trihydrate (1.0 g) was added and allowed to stir for 30 min. The reaction mixture was then poured into water and extracted twice with dichloromethane (CH_2_Cl_2_, 150 mL). The combined organic phase was dried over MgSO_4_, filtered and reduced in volume to approx. 10 mL, and then added dropwise to cold methanol (200 mL) under vigorous stirring. The precipitate was filtered through a cellulose thimble and purified by successive Soxhlet extractions with methanol, acetone and ethyl acetate. The residue was then extracted with chloroform and concentrated to approx. 5 mL and then added dropwise to cold methanol (200 mL) under vigorous stirring. The precipitate was then collected by filtration and dried in air and under high vacuum overnight to afford the product as an orange solid (201 mg; GPC: $M_{n}=33.8k,\;M_{w}=74.4k,\;PDI=2.2)$.

### 2.2 Preparation of films

cPFT2 and cPF were dissolved in toluene at a concentration of 30 mg/mL. Fused‐silica substrates were cleaned in an ultrasonic bath using acetone and isopropyl alcohol for 10 min each. They were then transferred to an oxygen plasma asher for 3 min at 80 W immediately prior to spin‐coating. Thin films were spin‐coated using a static dispense method at 1500 rpm for 60 s, using a Laurell WS‐650MZ‐23NPP spin coater. Thermal annealing was performed in a nitrogen glovebox, with <0.1 ppm H_2_O and O_2_, for 10 min at 140°C.

### 2.3 Characterization

CD spectra were measured using a Chirascan V100 spectrometer. Temperature‐dependent CD spectroscopy and Mueller matrix polarimetry were measured in transmission mode at the B23 beamline at the Diamond Light Source. In situ, temperature‐dependent spectra were obtained using a Linkam temperature‐controlled stage with nitrogen. The heating/cooling rate was 10°C/min and the thin films were held at a given temperature for 1 min prior to CD/MMP measurements. CD peaks for the spectra at each temperature were found via the find peaks algorithm in the scipy package for the python programming language.^29^ Photoluminescence was measured using an Edinburgh Instruments FLS 1000 spectrometer. Cross‐polarized microscopy was performed using a BX50 Olympus polarizing microscope. Atomic Force Microscopy was measured using an Asylum Instruments MFP‐3D in tapping mode. AFM images were processed using Gwyddion.^30^ Roughness parameters reported were calculated via Gwyddion and are the RMS ( $S_{q}$) parameter.

Film thicknesses were measured using a Dektak 3 profilometer by scratching the film and taking the mean of three measurements. DFT calculations were carried out using Gaussian 09 at the CAM‐B3LYP/6‐311G(d,p) level of theory.^31^, ^32^, ^33^, ^34^

## 3 RESULTS AND DISCUSSION

**FIGURE 2:**
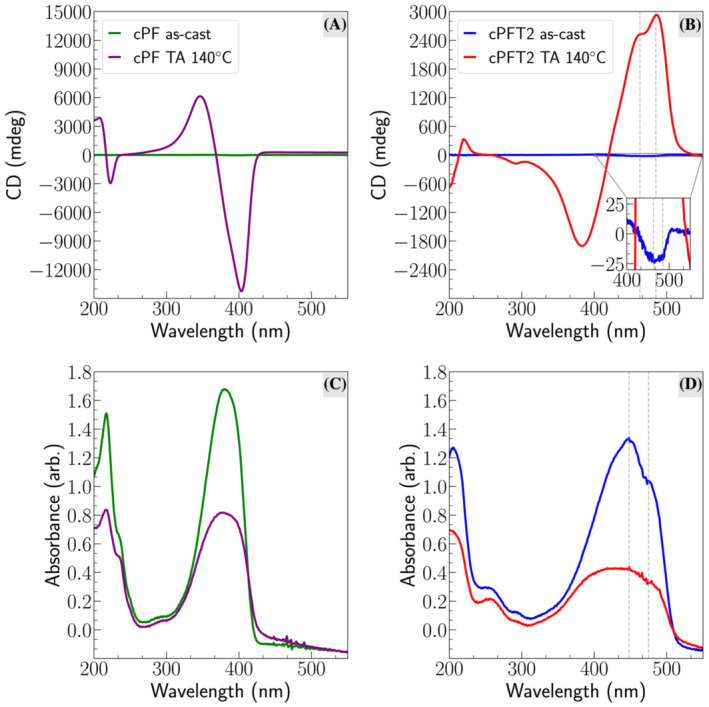
CD and absorption spectroscopy of annealed and as‐cast chiral side chain polymer films. (A) CD and (C) absorption spectra of as‐cast and annealed cPF, and (B) CD and (D) absorption spectra of as‐cast and annealed cPFT2. Dashed lines indicate the positions of vibronic features.

We have synthesized cPF and cPFT2 polymers using *(S)*‐3,7‐dimethyloctyl groups as chiral side chains for 9,9*′*‐disubstituted fluorene. Chiral polyfluorenes with these types of side chain have been previously shown to display giant chiroptical properties.^5^, ^14^, ^15^ For polyfluorene copolymers, it has previously been shown that the strongest chiroptical responses are generated when enantiopure side chains are attached to fluorene unit, with the size of the comonomer unit (torsional flexibility of the main chain) influencing the likelihood to form a chiral phase.^35^ We studied the photophysical and chiroptical properties of cPFT2 in solution (Figure S1) and thin films (Figure 2) via UV/vis and CD spectroscopy. cPFT2 demonstrates spectral features reminiscent of achiral F8T2.^36^ Both cPF and cPFT2 are CD silent in solution and demonstrate a weak optical activity in as‐cast films (Figure 2). Thermal annealing at 140°C results in a considerable chiroptical response, generating a pronounced bisignate Cotton effect that persists on cooling to room temperature, with maximum $g_{abs}$ of approximately 0.4 (500 nm) for cPFT2 and −0.7 (410 nm) for cPF (Figure S2). This is smaller than the $g_{abs}$ reported by our group for F8T2 blended with aza[6]helicene (1.3).^22^, ^23^ Interestingly, the sign of the CD is opposite for cPF and cPFT2, despite having the same handedness of chiral side chain, with cPF demonstrating an intense negative band at high wavelengths and a positive band at lower wavelengths, and cPFT2 the opposite. Notably, the sign of CD observed for cPFT2 resembles the CD spectra we previously reported in films of achiral F8T2 blended with *P*‐aza[6]helicene, while the CD observed for cPF here resembles the CD spectrum reported for achiral PFO blended with *M*‐aza[6]helicene.^22^, ^24^ This indicates that the chiral side chains have the opposite effect on the chiroptical response of cPF and cPFT2, which indicates differences in their supramolecular assembly and backbone conformation.

For cPFT2, the sign of the CD inverts upon annealing—the weak CD of the lowest energy transition in as‐cast cPFT2 (see inset of Figure 2) is negative, while the strong CD of the lowest energy transition in annealed cPFT2 is positive. A similar effect has been observed previously in chiral polyfluorenes, having been associated with the adoption of long‐range chiral order upon annealing.^15^ The line‐shape of the low energy transition in cPFT2 also changes: The as‐cast cPFT2 film exhibits strong absorption in the 350 – 500 nm region with a peak at ≈448 nm and a red‐shifted shoulder at ≈475 nm, while the annealed thin films show a large decrease in overall absorption intensity and intense CD in the long wavelength shoulder.

Previous studies of the achiral analog polymer F8T2 noted the appearance of an intense red‐shifted peak at ≈ 517 nm corresponding to the 0–0 vibronic peak upon annealing, attributed to the formation of tight J‐aggregate type structures with long range order.^36^ The absence of this peak at 517 nm in the absorption spectra indicates that chiral J‐aggregate type structures are not present in cPFT2 thin films. This is evidence that the dimethyloctyl side chains present on cPFT2 disrupt the usual aggregation behavior of F8T2.

**FIGURE 3:**
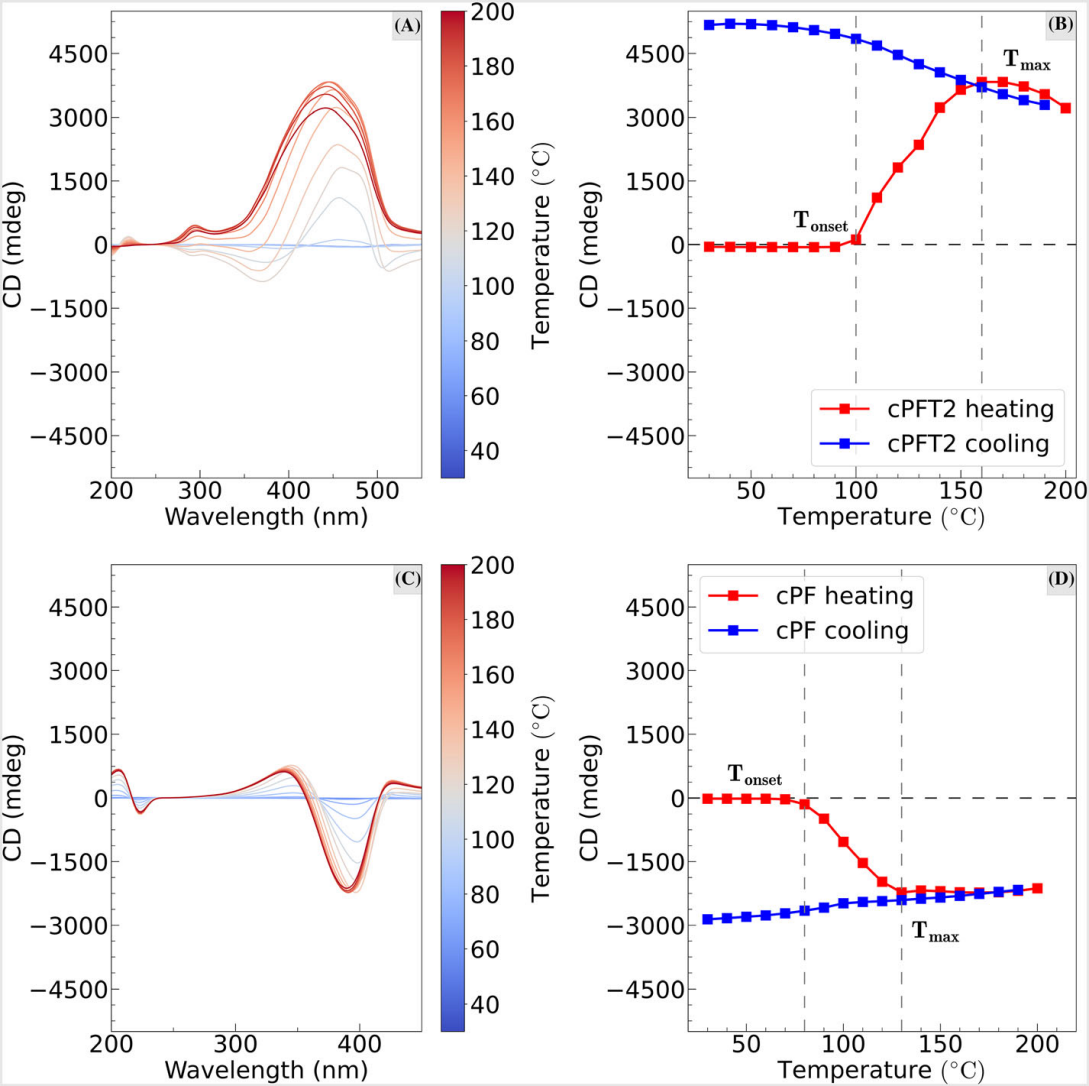
Results of in situ CD spectroscopy during heating and cooling of the two polymers under $N_{2}$. Dashed vertical lines in (B) and (D) indicate $T_{max}$ and $T_{onset}$ (see main text). (A) In situ CD spectra of a 98 nm thick cPFT2 film (20–200°C). Spectra acquired during the cooling cycle are included in the Supporting Information. (B) Peak CD of each spectrum versus temperature, for both the heating and cooling of the cPFT2 film. (C) In situ CD spectra of a 133 nm thick cPF film (20–200°C). (D) Peak CD of each spectrum versus temperature, for both the heating and cooling of the cPF film.

The change in optical activity due to annealing was studied using temperaturedependent synchotron‐radiation CD spectroscopy, shown in Figures 3 and S3–S8. The use of in situ CD offers insights into the self‐assembly processes. Above a certain temperature ( $T_{onset}$), both cPF and cPFT2 show a rapid increase in CD, until a maximum temperature ( $T_{max}$) after which the CD intensity decreases. $T_{onset}$ and $T_{max}$ for cPFT2 are slightly higher than cPF ( $T_{onset}$ cPF $\approx 80^{\circ }$C and cPFT2 $\approx 100^{\circ }$C, $T_{max}$ cPF $\approx 130^{\circ }$C, and cPFT2 $\approx 160^{\circ }$C) (Figure 3). We note that the $T_{onset}$ for cPFT2 observed roughly corresponds to the glass transition temperature measured by Joseph et al using DSC,^21^ and $T_{max}$ to the temperature of a peak in the DSC thermogram that the authors attributed to an irreversible phase transition to a more thermodynamically stable state than the kinetically stable as‐cast film. This indicates that in order to induce the formation of a chiral phase, heating above the glass transition is required. At higher temperatures around $T_{max}$, CD spectra of cPFT2 lose their bisignate line‐shape, and display a long wavelength shoulder. (Figure 3). Notably, this is the opposite to the behavior observed in films thermally annealed at 140°C and immediately quenched (Figure 2). The line‐shape does not recover upon cooling, but the magnitude of CD does. Further experiments are required to uncover the origin of this change, but it may be associated with the phase transition at a similar temperature observed by Joseph et al.^21^ We also examined the change in absorption as a function of temperature in cPFT2 (Figures S6 and S8) and cPF (Figures S3 and S4). During heating, the absorption intensity for both polymers significantly decreases with loss of the vibronic fine structure. The decrease of absorption intensity and broadening have been associated with a rearrangement of polymer backbones and increased chain coiling.^9^, ^27^, ^37^, ^38^

**FIGURE 4:**
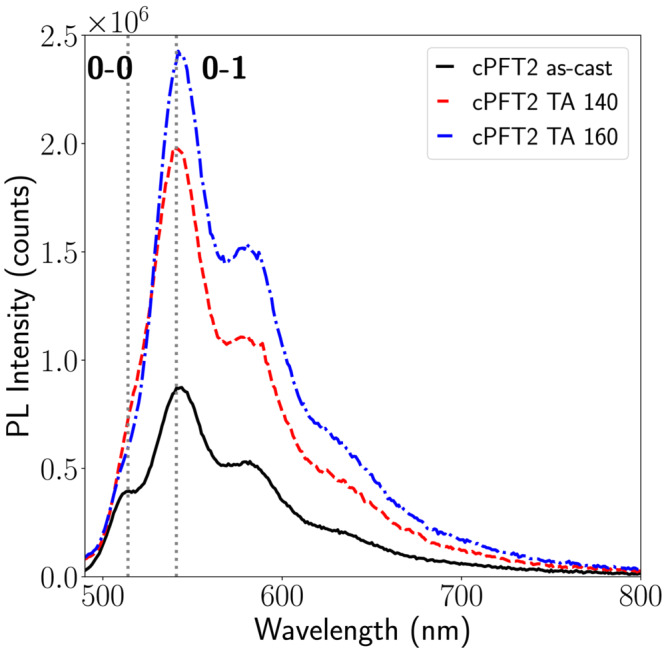
Photoluminescence spectra of cPFT2 films, as‐cast and annealed at $140^{\circ }$C and $160^{\circ }$C.

Photoluminescence (PL) spectra were acquired of as‐cast and annealed films of cPFT2 (Figure 4). The PL spectra include vibronic structure, with strong emission from the 0‐1 peak (λ=540 nm), and a weak 0‐0 shoulder (λ=514 nm). Upon annealing, the 0–0 peak decreases relative to the 0‐1 peak (Figure S9), which we explore in further detail in the Supporting Information. Hestand and Spano have shown PL is particularly sensitive to chain‐bending in conjugated polymers, and that chain bending is associated with with a decreased intensity of the 0–0 emission peak relative to the 0–1 peak.^39^, ^40^ The line‐shape of the PL spectra indicate that annealing cPFT2 increases backbone bending. A bent chromophore structure with *syn* thiophenes pointing inwards, and side chains pointing outwards, known as a helical cisoid, has been proposed as the origin of spectral features in achiral F8T2 aggregates and associated with the formation of single chain helices in other polythiophenes.^40^, ^41^ The assembly of cPFT2 chains into helical cisoid type structures via the in‐plane bending of the main backbone, with a handedness determined by the chiral side chain, may explain the difference in observed CD. Compared with cPF, this may be possible in cPFT2 due to greater conformational flexibility afforded by the more freely moving thiophene units and larger spacing between sterically hindering side chains. This theory is further supported by DFT calculations discussed below.

To further explore the origins of this chiroptical response, we used Mueller matrix polarimetry, which can discriminate between circular and linear contributions to the optical response (Figures S10 and S11). Logarithmic decomposition of the spectra of the homogeneously depolarizing film^42^ shows very small linear dichroism and birefringence, showing that the large CD observed is not apparent CD originating from the coupling of linear dichroism and birefringence.^10^, ^43^, ^44^, ^45^

**FIGURE 5:**
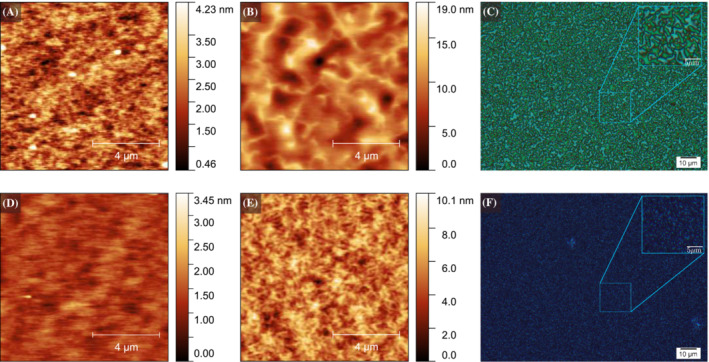
(A) AFM image of as‐cast cPFT2. (B) AFM image of annealed cPFT2. (C) Cross‐polarized microscope image of annealed cPFT2. (D) AFM image of as‐cast cPF. (E) AFM image of annealed cPF. (F) Cross polarized microscope image of annealed cPF.

Atomic Force Microscopy (AFM) provides information on the morphology and surface topography of the as‐cast and annealed films (Figure 5). Calculation of roughness parameters for each image (Table 1) shows that annealing increases the surface roughness for both cPF and cPFT2. The as‐cast cPF film is smooth and featureless, with small twisted fibrils (300 nm long and 20 nm wide) that appear after annealing. Similar fibril‐like structures are also present in the as‐cast cPFT2 films and extend into curved, connected domains upon annealing. The large differences in morphology support the theory that the thiophene units afford greater torsional flexibility in the backbone of cPFT2, which leads to a significantly different polymer microstructure after annealing. Polarized optical microscopy reveals that annealed films of both polymers comprise of micron sized crystalline domains, with cPFT2 showing larger, smoother domains with a more distinct texture.

**TABLE 1:** Roughess parameters.

Sample	RMS roughness (nm)
cPF as‐cast	0.23
cPF (annealed)	1.20
cPFT2 as‐cast	0.59
cPFT2 (annealed)	2.40

**FIGURE 6:**
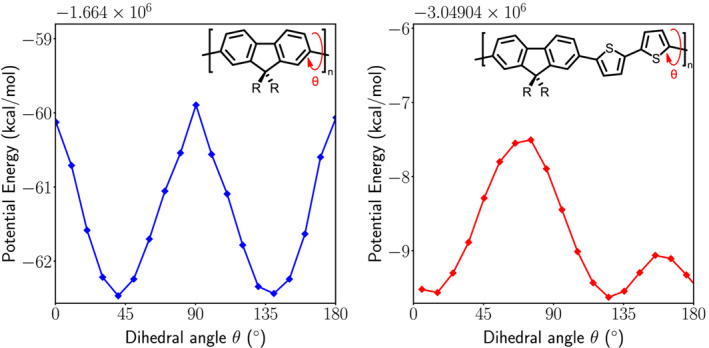
(R = *S*‐3,7‐dimethyloctyl) Relaxed potential energy scan of cPF and cPFT2 dimers. Dimers consisting of two of the repeating units in cPF and cPFT2, with the main chains capped by Me groups, were constructed using Gaussview, then the structure optimized using Gaussian 09 at the CAM‐B3LYP/6‐311G(d,p) level of theory. The dihedral angle highlighted with a red arrow on each diagram was changed, then the structure optimized again, keeping the dihedral angle fixed at this new angle. The process was repeated to scan the potential energy surface as a function of dihedral angle. The full side chain was included in the model.

**FIGURE 7:**
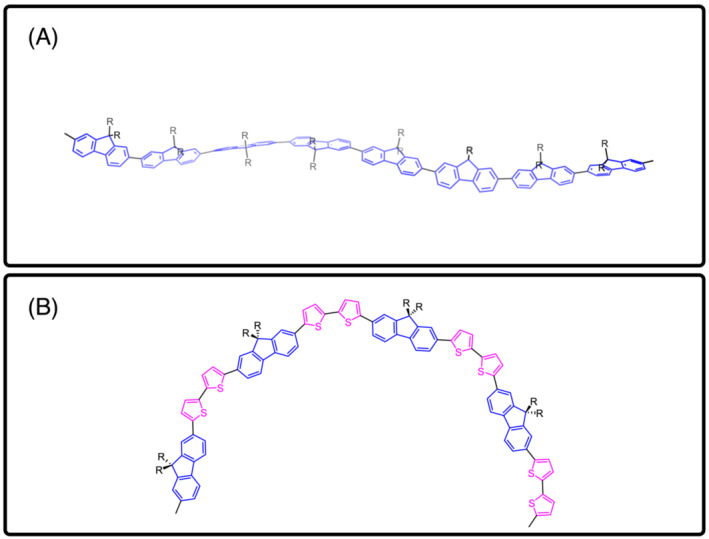
Cartoons representing potential polymer backbone conformations. (A) A straight main chain conformation, with a ribbon‐like twist along it. (B) The “bent” chromophore arrangement, in which the backbone adopts a coil‐like structure. We propose that cPF adopts a configuration like (A), while cPFT2 adopts the structure like (B).

Theoretical calculations withdensity functional theory at the CAM‐B3LYP/6‐311G(d,p) level for cPFT2 and cPF were performed using Gaussian 09 to gain insight into the molecular geometry of the backbone with and without the thiophene units, and the influence of the chiral side chain upon preferred conformation. As shown in Figure 6, a scan of the potential energy surface versus the dihedral angle between adjacent monomer units (θ) indicates differences in the preferred conformations of cPF and cPFT2 backbones. Energy minima at approximately $\theta =135^{\circ }$ appear in both systems, however interestingly, dihedral angles which place the (*S*)‐3,7‐dimethyloctyl side chains in a *syn* $\left(\theta =0^{\circ }\right)$ or *anti* $\left(\theta =180^{\circ }\right)$ conformation appear as energy maxima in the simulation of cPF, and as energy minima in cPFT2. These predictions indicate that cPFT2 polymer chains have a greater degree of planarity compared to cPF, and are stable with aliphatic side chains in a *syn* conformation, which when combined with the discussion of PL evidence above supports the proposal that cPFT2 chains adopt a bent‐chromophore type conformation upon annealing. This is contrasted with cPF, where the results show that such a backbone conformation would be strongly disfavored, with the polymer instead favoring the adoption of a straight main chain. These conformations are illustrated in Figure 7.

## 4 CONCLUSION

The chiroptical properties of chiral side chain polyfluorenes depend on the chemical structure of polymer backbone. We have compared the chiroptical properties and morphology of cPFT2 and cPF and identified a possible origin of the opposite sign CD. AFM reveals large differences in the self‐assembly processes of the two polymers. Comparison with the CD line‐shapes associated with chiral induction in F8T2 via other methods indicate that the *S*‐enantiomer side chains induce chirality in the polymer backbone in the opposite manner to which they do in cPF and other chiral polyfluorenes with shorter monomers. For example, previously reported blends of F8T2 with *P*‐aza[6]helicene match the sign of CD observed for cPFT2, while blends of PFO with *M*‐aza[6]helicene match that observed for cPF.^22^ However, the | $g_{abs}$| of cPFT2 is not as large as is observed in F8T2:aza[6]helicene blends. Photoluminescence studies indicate a degree of chain‐bending upon annealing providing evidence that suggests the adoption of a helical‐cisoid type polymer conformation, with outward facing chiral side chains influencing the turn direction. This is contrasted to cPF, in which tighter steric requirements restrict polymer flexibility and enforce a larger degree of nonplanarity, preventing coiling and leading to a different supramolecular structure. Further work is needed to understand the morphology of chiral side chain polyfluorenes and annealing induced changes in‐depth, but these results show that simple rules following from the enantiomer of side chain on a polymer backbone cannot reliably be used to predict the sign of the chiropitcal response of CD in thin films.

## Supporting information

chir23601supinfo‐0001‐supplementaryinfocF8T2.pdf

## Data Availability

Data are available upon request.
