# Supplementary material for: Insights into the origins of inverted circular dichroism in thin films of a chiral side chain polyfluorene
Source: Chirality. 2023 Jun 22;35(11):817–25. doi: 10.1002/chir.23601 (PMC10946986; doi:10.1002/chir.23601)
Supplement: Supplementary file 1 — chir23601supinfo‐0001‐supplementaryinfocF8T2.pdf [file CHIR-35-817-s001.pdf]

Supporting Information

Insights into the origins of inverted circular dichroism in thin films of a chiral sidechain polyfluorene

Louis Minion,[a,c,d] Jessica Wade,[a,c] Juan Manuel Moreno-Naranjo[b,c], Sean Ryan[b], Giuliano Siligardi[d], and Matthew J. Fuchter[b,c]

[a]*Department of Materials, Exhibition Road, Imperial College London, SW7 2AZ*

[b]*Department of Chemistry and Molecular Sciences Research Hub, Imperial College London, White City Campus, 82 Wood Lane, London W12 0BZ, UK.*

[c]*Centre for Processable Electronics, Imperial College London, South Kensington Campus, London SW7 2AZ, UK.*

[d]*B23 Beamline, Diamond Light Source Ltd, Harwell Science and Innovation Campus, Didcot, UK*

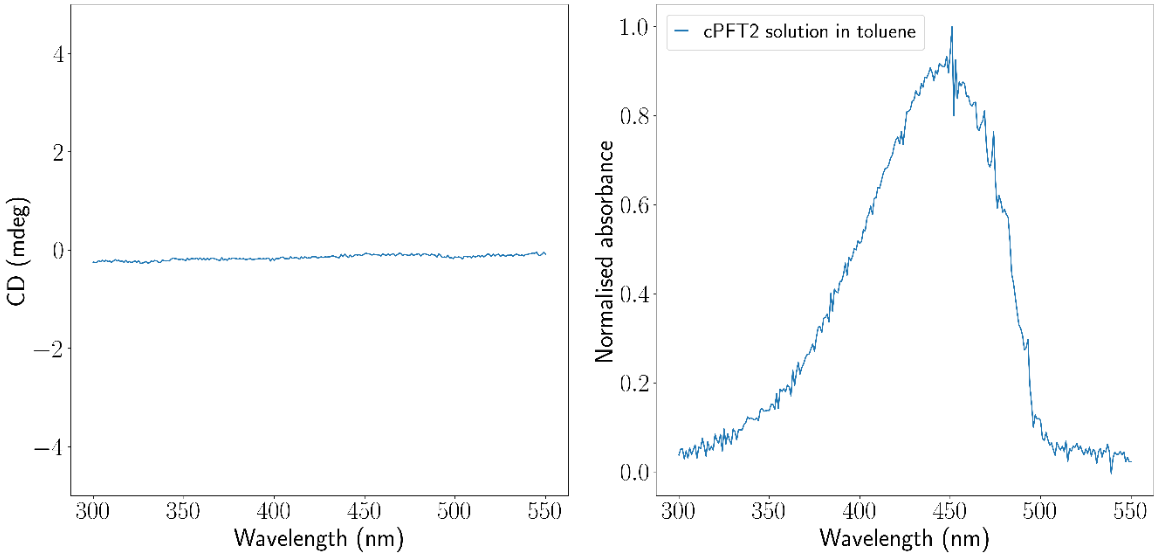

Supplementary Figure 1 CD and UV-Vis absorption of cPFT2 in toluene solution.

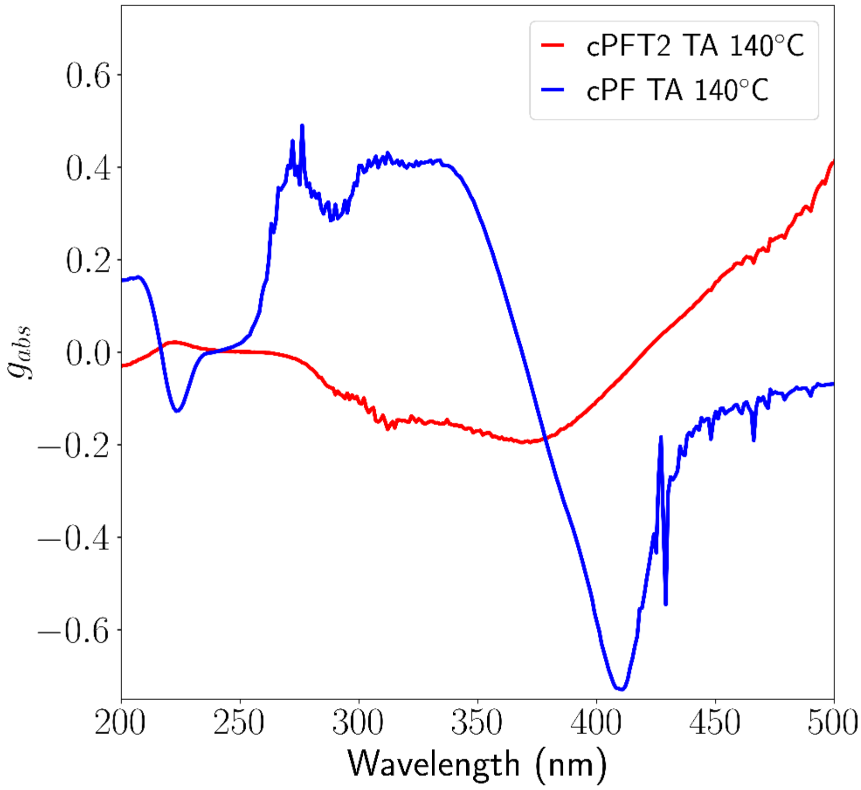

Supplementary Figure 2. g-factor of absorbance (CD/Abs) measured of thin films of cPF and cPFT2 annealed at 140C and rapidly quenched.

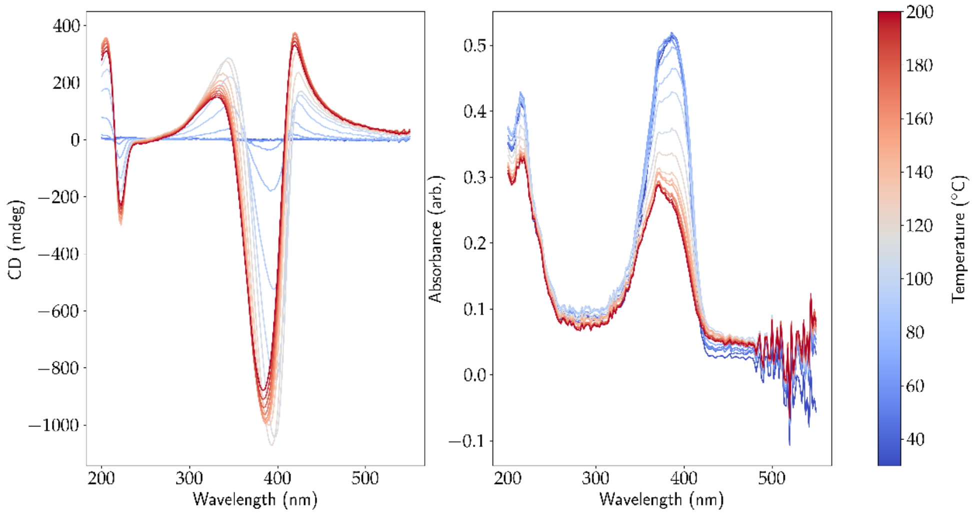

Supplementary Figure 3. CD and absorption spectra taken every 10C during heating to 200C of a second film of cPF (film 2), with film 1 CD spectra shown in Figure 3.

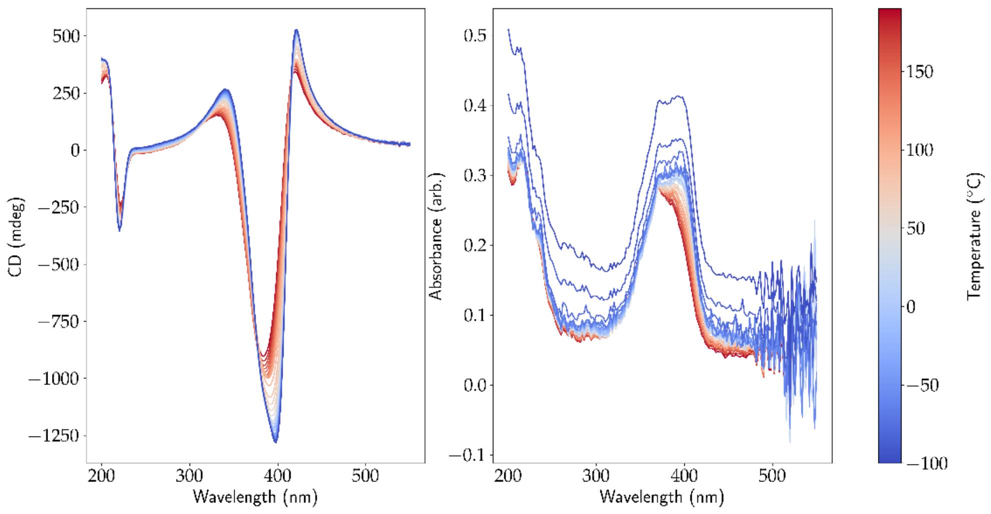

Supplementary Figure 4. cPF film 2 CD and absorption spectra taken every 10C during cooling from 200 C to -100C

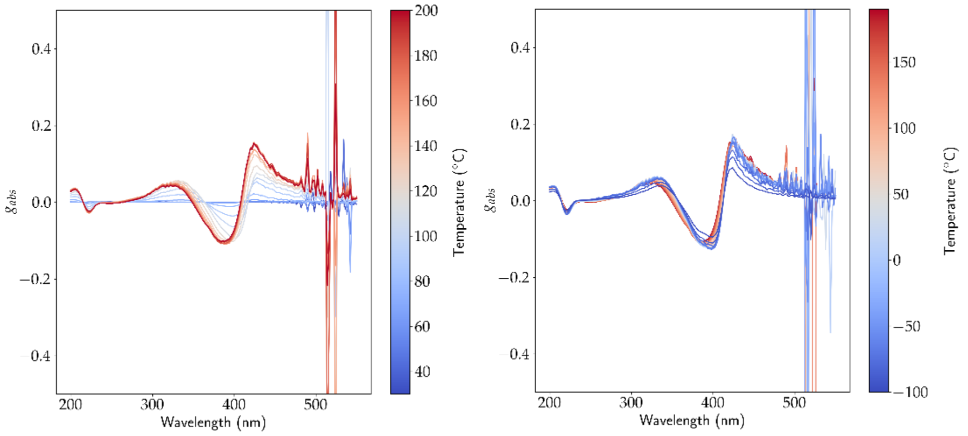

Supplementary Figure 5. Left; g-factor of absorption calculated from CD and absorption spectra during heating of cPF film 2 to 200C, right the same during cooling from 200 C to -100 C

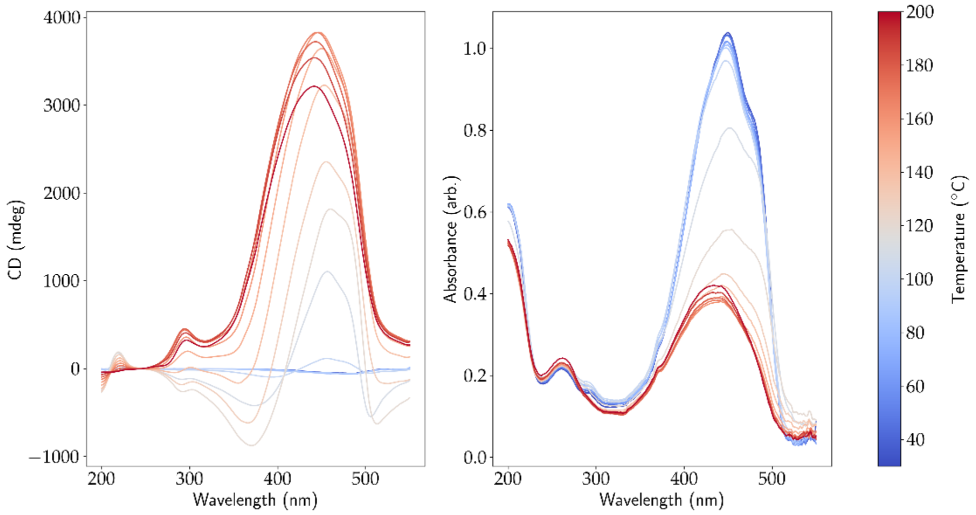

Supplementary Figure 6. Temperature dependent CD and absorbance of a cPFT2 film during heating to 200C.

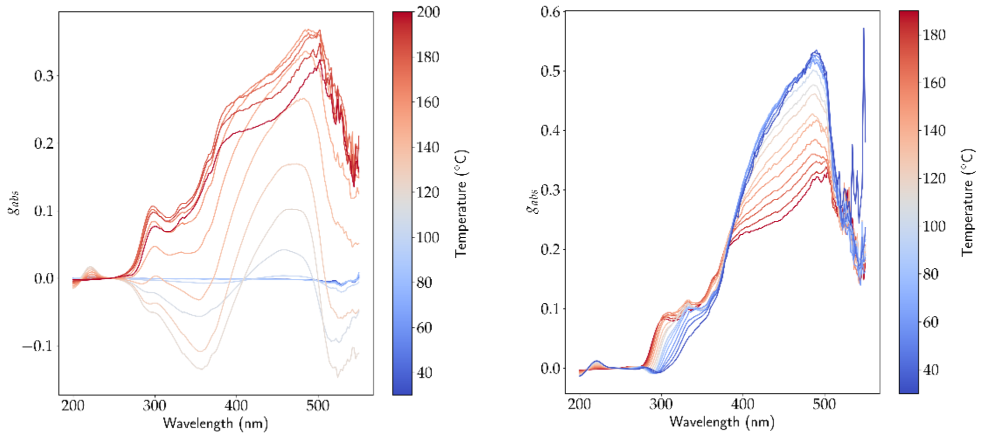

Supplementary Figure 7. g-factor of cPFT2 film during heating to 200C (left) and cooling to room temperature (right).

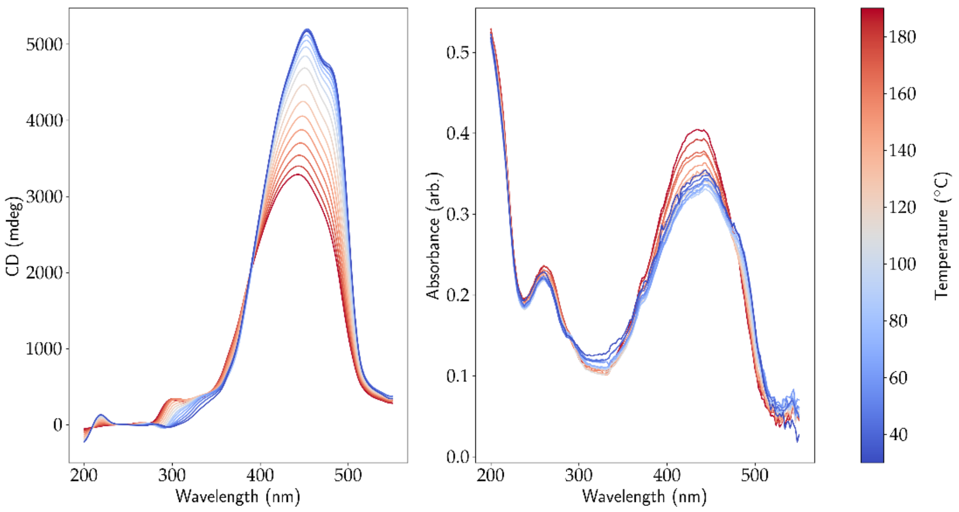

Supplementary Figure 8. Temperature dependent CD and absorption for cPFT2 film during cooling from 200C.

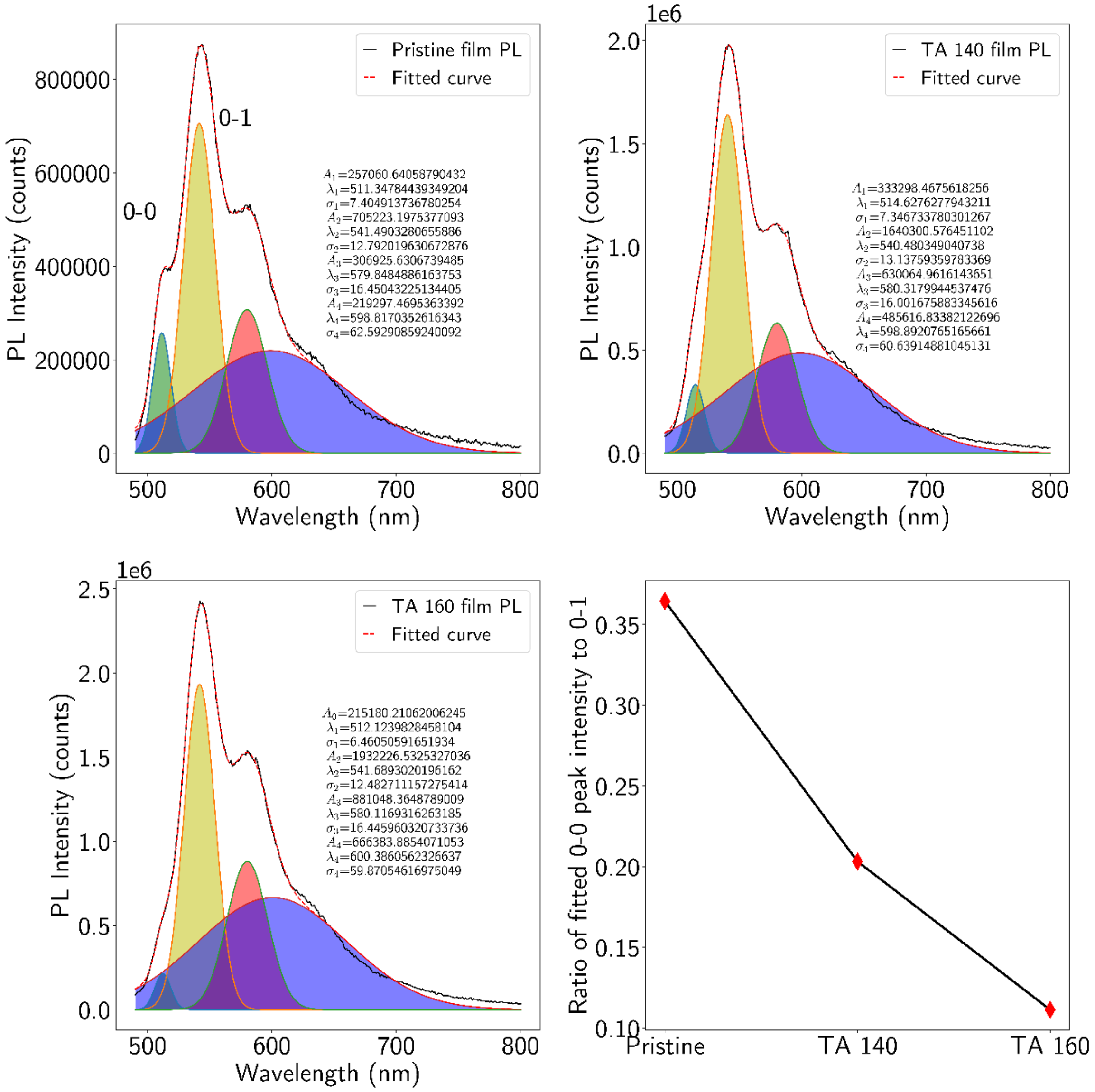

Supplementary Figure 9. Fitted PL spectra of cPFT2 films; pristine and annealed at two temperatures. Curve was fitted as the sum of four gaussians via the scipy python library, and values reported are the fitted parameters. Lower right graph shows the ratio of the fitted intensities of the two highest energy transitions (0-0 and 0-1) plotted versus cPFT2 film annealing condition, showing a significant drop.

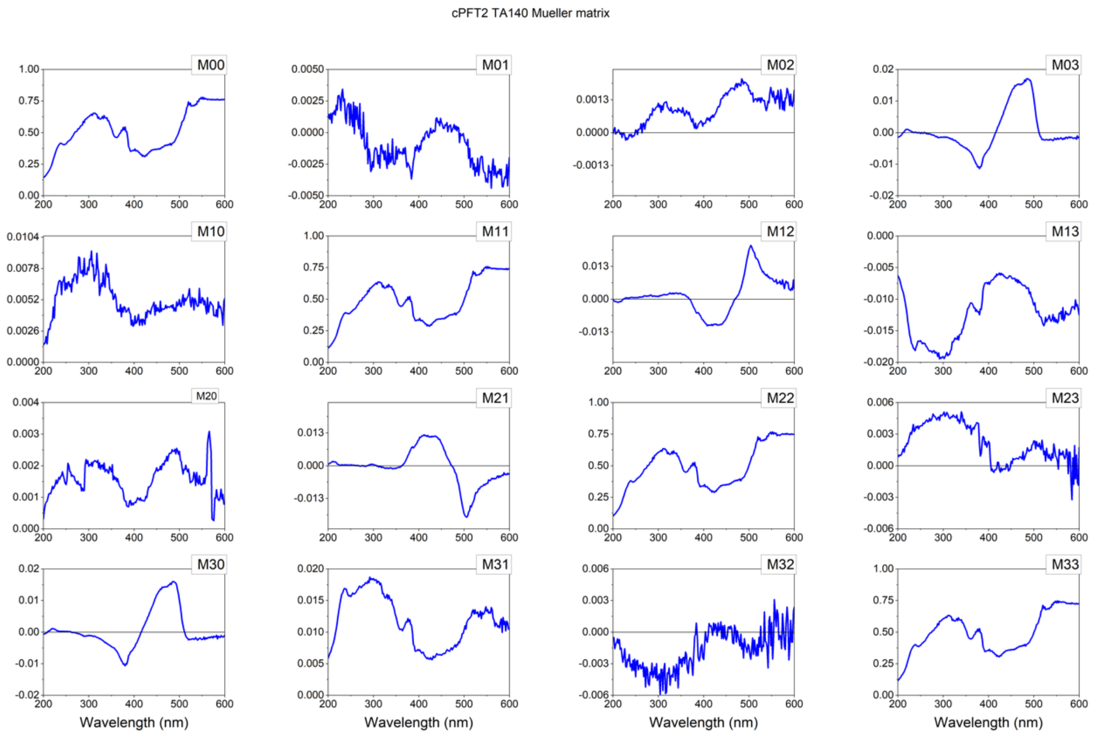

Supplementary Figure 10. Full transmission Mueller matrix of cPFT2 measured using the Mueller matrix polarimeter at the B23 beamline at the Diamond Light Source.

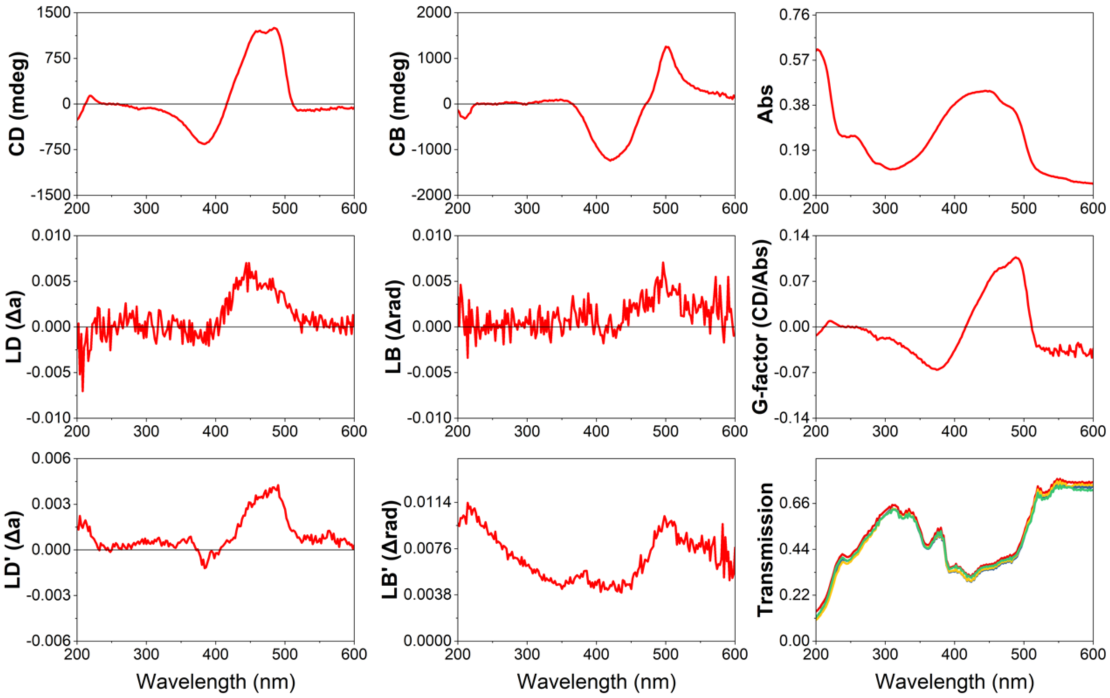

Supplementary Figure 11. Matrix logarithm decomposition of the transmission Mueller matrix of an annealed cPFT2 film, showing the 9 individual optical properties. Overlaid transmission spectra in the bottom right indicate spectra calculated from the m_{00}, m_{11}, m_{22}, and m_{33} elements of the full Mueller matrix.
